# MIQE 2.0 and the Urgent Need to Rethink qPCR Standards

**DOI:** 10.3390/ijms26114975

**Published:** 2025-05-22

**Authors:** Stephen Andrew Bustin

**Affiliations:** Medical Technology Research Centre, Anglia Ruskin University, Chelmsford CM1 1SQ, UK; stephen.bustin@aru.ac.uk

The recent publication of the revised MIQE 2.0 guidelines in Clinical Chemistry [1] marks a critical milestone in the evolution of quantitative real-time PCR (qPCR) methodology. These revised recommendations were authored by an international consortium of multidisciplinary experts in molecular biology, clinical diagnostics, statistics, regulatory science, and bioinformatics and build on the initial MIQE guidelines published in 2009 [2]. Over the past 16 years, MIQE has become one of the most widely cited methodological publications in molecular biology, with over 17,000 citations to date. It has helped to shape best practice in qPCR and reverse transcription-qPCR (RT-qPCR), informed journal editorial policies, and contributed to the development of ISO standards for molecular diagnostics [3].

The MIQE 2.0 guidelines take into account recent advances in qPCR technology and extend the original guidelines in several key areas. They explicitly explain why the whole qPCR workflow must adapt to emerging applications and provide coherent guidance for sample handling, assay design and validation, and qPCR data analysis. The reporting requirements, which are essential for transparency and reproducibility, have also been simplified and updated. Most importantly, they reinforce a simple but critical message: no matter how powerful the technique, without methodological rigour, data cannot be trusted.

As Editor-in-Chief of the Molecular Diagnostics section of IJMS, I adjudicate manuscripts with conflicting reviewers’ recommendations, and when qPCR data are included as part of the experimental evidence. In such cases, reviewer disagreement rarely concerns the qPCR data themselves, their biological interpretation or statistical thresholds. Nevertheless, an examination of the methods section generally reveals serious problems with the experimental workflow, ranging from poorly documented sample handling to absent assay validation, inappropriate normalisation, missing PCR efficiency calculations and nonexistent statistical justification. The result is often exaggerated sensitivity claims in diagnostic assays and overinterpreted fold-changes in gene expression studies. These failures are not isolated. They reflect a broader pattern that is abundantly evident in the published literature. Despite widespread awareness of MIQE, compliance remains patchy, and in many cases, entirely superficial [4].

This matters. It matters because qPCR is not a niche technique. It is arguably the most commonly employed molecular tool in life science and clinical laboratories. It matters because results derived from qPCR underpin decisions in biomedical research, diagnostics, pharmacology, agriculture, and public health. It matters because misinterpreted data carry real-world consequences. The COVID-19 pandemic demonstrated this with extraordinary clarity. While qPCR was central to global testing efforts, the variable quality of assay design, data interpretation, and public communication undermined confidence in diagnostics [5,6,7]. The situation is no better today: recent analyses of qPCR-based studies continue to reveal serious deficiencies in experimental transparency, assay validation, and data reporting [8,9,10].

It is essential to confront this reality with candour. There is a persistent—and troubling—complacency surrounding qPCR. While high-throughput sequencing and proteomic technologies are subjected to intense scrutiny, qPCR often escapes serious review. Nucleic acid quality and integrity are not properly assessed [11,12,13]. Fold-changes of 1.2- or 1.5-fold are routinely reported as biologically meaningful, even at low expression levels, without any assessment of measurement uncertainty or technical variance [14,15]. Genes are declared upregulated or downregulated with confidence intervals spanning thresholds of significance [16,17]. Assay efficiencies are assumed, not measured [18,19,20]. Normalisation is based on reference genes that are neither stable nor validated [21,22,23]. These are not marginal oversights, they are fundamental methodological failures.

Particularly in the context of molecular diagnostics, where qPCR is often used to infer pathogen load, expression status, or treatment response, such failures are unacceptable [24]. A diagnostic platform that cannot reliably distinguish a small fold change in low target concentration at clinically relevant levels is not fit for purpose. A method that fails to account for variability in reverse transcription, sample input, or reagent efficiency cannot support precise quantification. And yet these issues remain largely unaddressed in the majority of published work.

MIQE 2.0 offers a timely, authoritative, and detailed guide to remedying these deficiencies. But guidelines alone are not enough. What is needed now is cultural change—among researchers, reviewers, journal editors, and regulatory agencies. We must stop treating qPCR as a “black box” technology and instead apply the same expectations for transparency, validation, and reproducibility that are demanded of other molecular techniques.

This is not merely an academic issue. The dismantling of disease surveillance systems and the devaluation of evidence-based public health policy, most notably under the current administration in the United States, loudly broadcasts the societal costs of unreliable molecular data. When decisions about disease containment, treatment, or policy are based on flawed diagnostics, the consequences are measured in lives, not *p*-values.

To those who argue that rigorous implementation of MIQE slows down publication or complicates experimental design, the response is simple: if the data cannot be reproduced, they are not worth publishing. The purpose of scientific communication is not speed, but clarity, reliability, and truth. MIQE 2.0 provides a framework for achieving that in qPCR. It is now incumbent on all of us to make it a standard, not in name, but in practice.

The metaphor often applied to climate change is apt here: everyone agrees it is a problem, but no one wants to change their behaviour. The same is true for qPCR. We acknowledge its limitations, cite MIQE in our methods, and then proceed as though quality control is someone else’s responsibility. That must change.

We have the tools. We have the evidence. We now have the updated guidelines. What we need is the collective will to ensure that qPCR results are not just published, but are also robust, reproducible, and reliable. The credibility of molecular diagnostics, and the integrity of the research that supports it, depends on it.
